# Influence of Propolis Extract (Caffeic Acid Phenethyl Ester) Addition on the *Candida albicans* Adhesion and Surface Properties of Autopolymerized Acrylic Resin

**DOI:** 10.1155/2022/6118660

**Published:** 2022-05-04

**Authors:** Khalifa S. Al-Khalifa, Mohammed M. Gad, Faris A. Alshahrani, Firas K. Alqarawi, Faisal R. Hassanein, Zohaib Khurshid, Khalid S. Al-Abidi

**Affiliations:** ^1^Department of Preventive Dental Sciences, College of Dentistry, Imam Abdulrahman Bin Faisal University, Dammam 31441, Saudi Arabia; ^2^Department of Substitutive Dental Sciences, College of Dentistry, Imam Abdulrahman Bin Faisal University, Dammam 31441, Saudi Arabia; ^3^Dental Internship Program, College of Dentistry, King Abdulaziz University, Jeddah, Saudi Arabia; ^4^Department of Prosthodontics and Dental Implantology, College of Dentistry, King Faisal University, Al-Ahsa 31982, Saudi Arabia

## Abstract

**Background:**

Denture stomatitis has been linked to the adhesion and proliferation of *Candida albicans* (*C. albicans*) on denture bases, which is a common and recurrent problem in denture wearers. The current study aimed to evaluate the effect of incorporating caffeic acid phenethyl ester (CAPE) into autopolymerized polymethyl methacrylate (PMMA) acrylic resin on *C. albicans* adhesion, surface roughness, and hardness as well as the correlation between tested properties.

**Methods:**

Autopolymerized acrylic resin discs (*N* = 100, 50/*C. albicans* adhesion; 50/*C. albicans* surface roughness and hardness test) were fabricated in dimensions 15 × 2.5 mm, samples were categorized into 5 groups (*n* = 10) based on CAPE concentrations; unmodified (control), 2.5, 5, 10 and 15% wt of acrylic powder. Specimens were stored in distilled water for 48 h at 37°C. *C. albicans* adhesion was evaluated via direct culture method. Profilometer and Vickers hardness tester were used for surface roughness and hardness measurement. Post hoc Tukey's HSD with ANOVA test was performed to compare the difference of means amongst groups. *P* values were statistically significant at ≤0.05.

**Results:**

The addition of 2.5% of CAPE to PMMA has significantly reduced *C. albicans* counts in comparison to higher CAPE concentrations (*p* < 0.001). As for surface roughness, it was noticed that it increased with increased CAPE concentrations (*p* < 0.0001). While surface hardness decreased as CAPE concentrations increased (*p* < 0.0001). All tested properties showed a significant difference amongst groups for *C. albicans* colony count and surface parameters.

**Conclusion:**

The addition of 2.5% of CAPE to PMMA acrylic resin significantly decreased *C. albicans* count compared to higher CAPE concentrations. CAPE can be used as an adjunct in the prevention of DS by incorporating in the PMMA acrylic resin.

## 1. Introduction

Denture stomatitis (DS) is a highly prevalent condition that affects mainly the palatal mucosa in complete or partial denture wearers [1, 2]. Its clinical manifestation appears as erythematous diffused or localized patches underneath the prosthesis [2–5]. DS development may be related to multiple factors such as ill-fitting dentures, poor oral hygiene and fungal infection especially with *Candida albicans (C. albicans*) [6–8]. About 30–75% of denture wearers have experienced DS, even after antifungal therapy there was still a high recurrence rate [9–11].

The porous nature of denture base material creates the proper environment for the colonization and adherence of *C. albicans* to these porous surfaces. Biofilm formation and adhesion play a major role in increasing antifungal therapy resistance, and reducing cleansing efficiency required to eliminate biofilms [7, 8].

Studies have investigated the effect of mixing natural products with the denture base to evaluate their effect on inhibiting the growth *C. albicans* [12, 13]. One study assessed the addition of 1% (concentration) of henna powder to denture base materials that significantly reduced the count of *C. albicans* [12]. Another study assessed the addition of 0.5% of thymoquinone concentration with denture base materials, which also showed a significant reduction in the count of *C. albicans* [13].

Propolis has shown to be effective against fungal [14] and bacterial infections [15, 16], as well as their ability to fight inflammation [17] and is considered an antioxidant [18, 19].

Caffeic acid phenethyl ester (CAPE) is one of the active components of propolis which has been widely studied [20]. CAPE possesses a wide range of biological activities, including but not limited to antibacterial and antiviral effects [20]. Previously conducted CAPE studies have demonstrated effective antifungal action against *C. albicans* [21]. It has been also shown to inhibit *C. albicans* hyphal growth and bioﬁlm formation [22].

Minimum inhibitory concentration (MIC) is considered to be the lowest therapeutic level required from a medical agent, in which it inhibits both the development and growth of microorganisms [23, 24]. A study by Barrientos et al. [25] found that the MIC of propolis ranging from 0.90 to 8.22 g·mL^−1^ was able to inhibit the growth of mutans streptococci.

The therapeutic effect of CAPE in the MIC against *C. albicans* on denture base materials has not been extensively explored as a possible method for the prevention of DS. The aim of the current study was to evaluate the antifungal activity of CAPE-incorporated autopolymerized polymethyl methacrylate (PMMA) acrylic resin on *C. albicans*'s adhesion to PMMA, and its effect on PMMA's hardness and surface roughness. The null hypothesis states that CAPE-incorporated autopolymerized acrylic resin has no effect on *C. albicans* adhesion, surface roughness, and hardness.

## 2. Materials and Methods

The sample size for the study was calculated according to previous studies [26–29]. The sample size was designed to be 10 for each group, with a 95% confidence interval and a minimum power of 80%, which lead to a total of 100 discs (50/*C. albicans* adhesion and 50/*C. albicans* surface roughness and hardness) specimens were required.

### 2.1. PMMA/CAPE Mixture Preparations

CAPE powder (100% purity from Biosynth Carbosynth Ltd. Compton, UK) was weighted using a digital balance in different concentrations of 2.5% wt, 5% wt, 10% wt, and 15% wt of autopolymerized acrylic resin powder (Major repair; Prodotti Dentari SPA, Italy). Concentrations were decided based on a pilot study result, which evaluated the minimum inhibitory effect of the CAPE concentrations used from 0.05% to 20%. Each concentration was thoroughly mixed starting with hand mixing followed by using an electric mixer till homogenous distribution of CAPE within the acrylic powder.

### 2.2. Acrylic Resin Specimens' Preparations

Acrylic resin specimens were prepared according to the different CAPE concentrations of 2.5% wt, 5% wt, 10% wt, and 15% wt, while, a fifth group was unmodified to serve as a control group. A negative metal mold with an internal dimension (15 × 2.5 mm) with corresponding metal cover was prepared and used for specimens' preparation. According to manufacturer instruction, the polymer/monomer ratio was prepared and mixed and then packed in the mold spaces under pressure and then placed in pressure put under pressure (30 lb/in^2^) for 15 minutes at 40°C. After complete polymerization, the retrieved specimens did not achieve complete polymerization; therefore, additional polymerization cycles at a higher temperature (65°C) were required. Specimens' excess resins were finished starting with excess resin removal using tungsten carbide bur and followed by standard polishing with a polishing machine (Metaserve 250 grinder-polisher; Buehler, Lake Bluff, IL) as described in previous studies [26–29]. Finally, the specimens were evaluated to be within accurate dimensions and free from surface irregularities and porosities. The final specimens were stored in sterile distilled water of 37°C for 48 h for the complete removal of residual monomers.

### 2.3. Microbiology Test

#### 2.3.1. Microorganism and Media

*C. albicans.* (ATCC10231) was used in this research, obtained from Microbiologics, St. Cloud, Minnesota, USA. They were cultivated on Sabouraud's dextrose agar (HiMedia Laboratories Pvt. Ltd., India) for 48 h at 37°C. After this period, *C. albicans* colonies were picked up from the new culture and suspended in 2 ml of sterile phosphate buffer containing 2 × 10^6^ cells.

#### 2.3.2. Microbiology Test

*(1) Exposing Acrylic Specimens with Different Concentrations of CAPE to C. albicans*. Ethyl alcohol (70% concentration) was used to sterilize the acrylic specimens and the specimens were cleaned with an ultrasonic machine with distilled water before their use in the experiments. The sterilized acrylic specimens with different concentrations of CAPE and another without (control) were soaked in 2 ml of sterile phosphate buffer containing 2 × 10^6^ cells for 48 hours at 37°C.

*(2) Isolation of Adherent C. albicans cells*. To detach nonadherent cells, all acrylic plates were rinsed 3 times, using sterile phosphate-buffered saline (PBS). The amount of *C. albicans* attached to acrylic specimens was determined by immersing the acrylic plates into tubes that contained 1 ml of Sabouraud dextrose broth for 24 hours. Then, the plates were vibrated with a vortex mixer for 10 minutes. These tubes were centrifuged for 5 minutes at 4500 rpm to obtain clustered pellets of *C. albicans*. Afterwards, concentrated pellets were gathered from their tubes after the acrylic resin plates were removed.

*(3) Antifungal Activity Assay of CAPE Against C. Albicans*. In order to calculate the quantity of live *C. albicans* for each sample, 0.1 ml of each bullet was taken, mixed with 0.9 ml sterile Sabouraud dextrose broth, and was diluted serially. Then, 250 *µ*l were obtained from each concentration and was cultured on a Sabouraud dextrose agar (SDA) and incubated at 37°C for 48 h. Triplicates of the CAPE and control were completed in the study.

*(4) Minimum Inhibitory Concentration (MIC) Test*. After an incubation of 48 hours, the quantities of colonies forming units/millimeter were calculated for each concentration using a Colony Counter (Bel-Art Scienceware, Wayne, NJ, USA). This would detect the lowest CAPE inhibitory concentration (MIC) that is required to prevent fungal growth [24].

### 2.4. Surface Roughness

An optical-noncontact-profilometer (Contour GT; Bruker Nano gmbH, Schwarzschildstrasse, Berlin, Germany) was used to measure the specimens' surface roughness. An area of 0.43 × 0.58 mm of each specimen was scanned with a standard camera at three sites under a magnification of 20×, and then, the mean *R*_a_ was calculated.

### 2.5. Hardness

Hardness values were obtained with a Vickers tester (Wilson Hardness, ITW Test & Measurement GmbH, Shanghai, China), A 25-gf load diamond indenter was applied perpendicular to each sample for 15 s, where hardness values were recorded digitally. The average was based on three readings for each sample.

### 2.6. Statistical Analysis

Kolmogorov–Smirnov test of normality was applied to assess the normality of distribution. To evaluate the impact of different concentrations of CAPE on *C. albicans* colony counts and surface properties, an ANOVA test was used followed by post hoc pairwise comparisons, in accordance with Dunnet and Scheffe. The Pearson correlation coefficient calculated the association level between *C. albicans* colony numbers and the various surface parameters. A two-tailed test was used with a *p* ≤ 0.05 considered statistically significant.

## 3. Results

**Figure 1:**
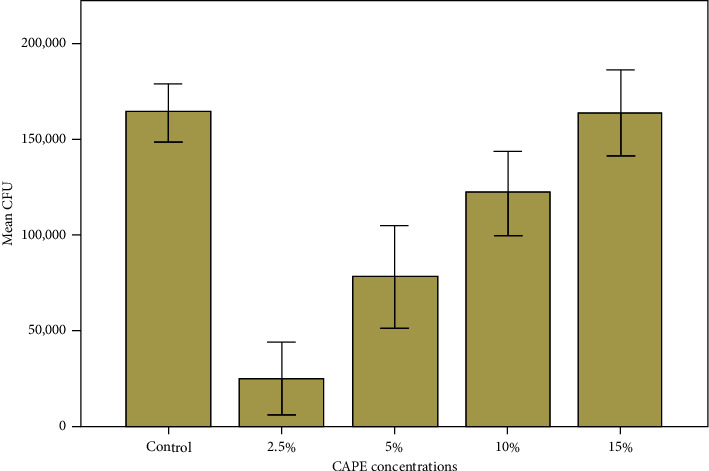
Effect of CAPE concentrations on denture acrylic base presented by mean CFU/mL of *C. albicans*.

*C. albicans* colony count ranged between 25,000 CFU/mL to 163,000 CFU/mL where ANOVA analysis revealed significant differences amongst tested groups (*F* = −35.962, *P* < 0.0001). Results of the *C. albicans* colony count from test groups using CFU are presented in Figure 1. In comparison to control, CAPE addition significantly decreased *C. albicans* colony counts (*P* < 0.001) except for the CAPE 15% group (*P*=0.99). In between CAPE groups, as CAPE concentration increased, *C. albicans* colony count increased as well.

**Figure 2:**
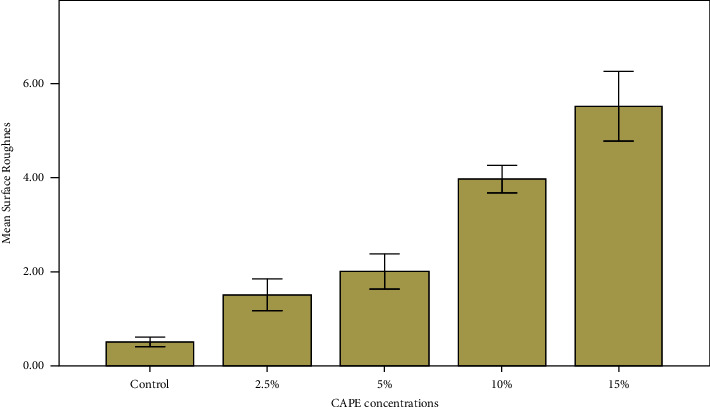
Effect of CAPE concentration on denture acrylic base presented by mean surface roughness (*µ*m).

Results of surface roughness (Ra) from test groups are presented in Figure 2. As CAPE concentration increased, surface roughness increased as well. Surface roughness ranged between 1 and 6 *µ*m and the mean difference amongst groups was statistically different (*P* < 0.0001).

**Figure 3:**
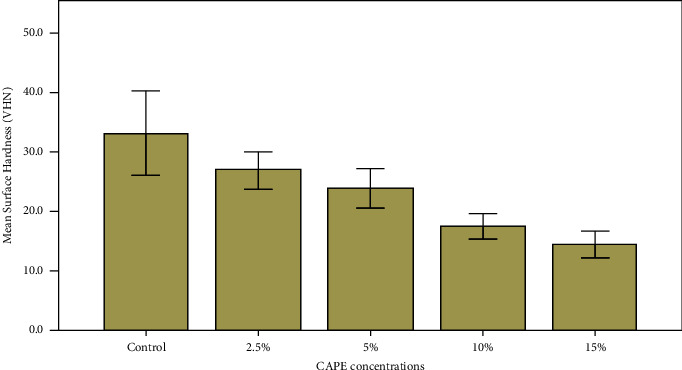
Effect of CAPE concentration on denture acrylic base presented by mean surface hardness (VHN).

Results of the acrylic discs surface hardness (VHN) in the various test groups are presented in Figure 3. As CAPE concentration increased, surface hardness decreased. Surface hardness ranged between 27 and 14 (VHN), and the mean difference between the groups was statistically different (*P* < 0.0001).

In Table 1, the mean, standard deviation (SD) and significances amongst groups for all tested properties according to CAPE concentrations are presented. All tested properties showed a significant difference between the groups for *C. albicans* colony count and surface parameters.

Levels of association between the *C. albicans* colony count and various surface parameters are shown in Table 2. Pearson correlation analysis revealed a positive correlation between *C. albicans* colony count and surface roughness while the correlation was negative between *C. albicans* colony count and surface hardness. Furthermore, a negative correlation was found between surface roughness and surface hardness which was highly significant.

## 4. Discussion

The aim of the current study was to investigate whether CAPE has the potential to reduce the adherence of *C. albicans* to autopolymerized acrylic resin. This in turn would help in preventing DS, which frequently occurs in complete dentures patients [30]. Also, to assess the effect of CAPE on surface properties of autopolymerized acrylic resin. The null hypothesis was rejected as CAPE addition to autopolymerized resin affected *C. albicans* adhesion and surface roughness and hardness.

The results showed that the addition of 2.5% of CAPE to PMMA significantly decreased *C. albicans* count compared to higher CAPE concentrations. It was also found that with increased CAPE concentrations, a noticeable increase in surface roughness existed. On the other hand, a decreased surface hardness of autopolymerized acrylic resin was observed with higher CAPE concentrations.

Previous studies have looked into CAPE's medicinal effect on different dental procedures. A study by Günay et al. [31]. evaluated the outcomes of CAPE on defects related to the palatal mucosa and extraction sockets where socket healing was improved significantly. Other studies evaluated CAPE's antibacterial effects on oral cariogenic bacteria. These studies showed a remarkable antimicrobial effect on cariogenic bacteria by inhibiting biofilms formation [32, 33].

Previous studies investigated the effect of mechanically and chemically cleaning removable prostheses, they have found that both methods could not eliminate adequately the contaminating microorganisms [34–36]. Due to an increase in antimicrobial resistance, studies have focused on evaluating new antifungal agents [37–41]. Our results are similar to another study that investigated the CAPE incorporation within the materials used for denture base fabrication [42].

Previous investigations have documented that surface roughness proportionally increase the attachment of microorganism to denture base materials [43, 44]. Results of the present study have shown that Ra values were significantly higher as the concentration levels of CAPE were increased.

The rougher the denture base surfaces were the more colonization and adhesion of *C. albicans* occurred [45]. Microbial adhesion and plaque accumulation are impacted by the denture base material's surface roughness [46, 47]. Rough denture base surfaces offered microbes more surface areas for adhesion. Rough surfaces aided in trapping microorganisms, this made cleaning dentures ineffective, even when antimicrobial agents were used [48]. This was noticed in our study, where with higher CAPE concentrations there was increased colonization of *C. albicans*, similarly rough surfaces proportionally increase the colonies in modified groups.

Hardness values were reduced and it was related to the additive materials in the PMMA denture base, this was due to their impact on the polymer matrix's integrity [44]. These have also been shown to cause stress within the polymer matrix [45, 46]. CAPE addition acted as an impurity, and to a weaker bonding process of CAPE to the resin matrix of acrylic denture bases, this was mainly because of the negative effect on conversion degrees required for a between bond. It has also shown increased levels of unreacted residual monomer, which made them act as a plasticizer [47, 48].

It has been reported that DS could be further prevented when CAPE was used with PMMA, even though this addition resulted in physical properties alterations. This was similar to another study [12] where a 0.5% natural henna concentration level was reported as the acceptable level to be added to PMMA, while higher levels of natural henna concentrations resulted in poor physical properties.

### 4.1. Clinical Significance and Clinical Recommendations

Clinically, CAPE could be recommended for incorporation into PMMA denture base material as an antifungal agent for DS treatment. Moreover, it could be added to hard and soft liners and all interim removable prostheses.

The limitations of this study were that no simulated aging procedure was conducted and the experiment was on one microorganism not involving other possible oral pathogens. Future studies such as surface treatments, and aging tests such as thermal cycling, immersion in water, and antifungal long-term effects would be recommended.

## 5. Conclusion

Within the limitations of the study, the addition of 2.5% of CAPE to PMMA acrylic resin significantly decreased *C. albicans* count compared to higher CAPE concentrations. CAPE can be used as an adjunct in the prevention of DS by incorporating it in the PMMA acrylic resin.

## Figures and Tables

**Table 1:** Mean, standard deviation (SD) and significances between groups for all tested properties according to CAPE concentrations.

Properties	Control	CAPE groups and % (mean ± SD)				ANOVA	
		2.5%	5%	10%	15%	*F*	*P* value
*C. albicans* count (CFU/mL)	163857.1 ± 15366.9	25333.3 ± 18929.7	78250.0 ± 26687.3	122142.9 ± 21851.2	163625.0 ± 22481.3	35.9	<0.0001
Surface roughness (*µ*m)	0.5 ± 0.1	1.5 ± 0.3	2.0 ± 0.4	3.9 ± 0.3	5.5 ± 0.7	200.4	<0.0001
Hardness (VHN)	33.2 ± 7.1	26.9 ± 3.2	23.9 ± 3.3	17.5 ± 2.1	14.4 ± 2.3	20.4	<0.0001

**Table 2:** Pearson correlation coefficients between *C. albicans* colony count and surface parameters.

	Surface roughness	Surface hardness
CFU	*r* = 0.263	*r* = −0.127
	*p*=0.139	*p*=0.554
Surface roughness		*r* = −0.832
		*p* < 0.0001^*∗*^

## Data Availability

The data used to support the findings of this study are available from the corresponding author upon request.
